# Functional recovery and quality-of-life improvement 12 months after LVAD implantation: A single-center cohort

**DOI:** 10.1016/j.jhlto.2026.100633

**Published:** 2026-07-14

**Authors:** Mohammed Najdat Seijari, Taraneh Zamani, Ma'in Abumuhfouz, Souwdamini Sethuram, Mohamed Fael, Saadeddine Khayat, Angel Mena, Peter Dinh Tran, Kathryn Lynne O'Keefe, Omar H. Jawaid, Samer Kaspo, Ahmed Chaaban, Sateesh Kesari

**Affiliations:** a TriHealth Good Samaritan Hospital, Cincinnati, OH; b TriHealth Heart & Vascular Institute, Cincinnati, OH; c TriHealth Good Samaritan Hospital, Infectious Diseases Department, Cincinnati, OH; d University of Missouri - Kansas City (UMKC), Kansas City, MO; e Saint Francis Hospital, Chicago, IL; f University at Buffalo, New York, NY

**Keywords:** Left ventricular assist device, HeartMate 3, Functional capacity, Quality of life, Patient-reported outcomes, Advanced heart failure

## Abstract

**Background:**

Left ventricular assist devices (LVADs) improve survival in advanced heart failure, but the extent of functional and quality-of-life (QoL) recovery remains incompletely characterized.

**Methods:**

This single-center retrospective study evaluated 41 LVAD recipients at baseline and 12 months post-implant in a contemporary community-based program, with 95% of implants performed as destination therapy, using standardized instruments (KCCQ-12, EQ-5D-3L, EQ-VAS, 6MWD). Paired statistical testing assessed within-patient changes.

**Results:**

KCCQ-12 scores increased from 36.1 ± 11.0–46.1 ± 10.8 (p < 0.001); 6MWD improved from 242 ± 104 m to 320 ± 113 m (p = 0.0054); and EQ-VAS improved from 54.4 ± 23.0–72.6 ± 17.0 (p < 0.001). Domains reflecting enjoyment of life (p = 0.025), ability to perform household chores (p = 0.026), and jogging/hurrying limitation (p = 0.018) all improved significantly. Self-reported stress decreased (6.4 → 4.4; p = 0.002), and satisfaction with HF therapy increased (7.2 → 8.5; p = 0.033).

**Conclusions:**

One year after LVAD implantation, patients demonstrated improvements in functional capacity and QoL, underscoring the importance of integrating psychosocial and rehabilitative strategies in LVAD care.

## Introduction

LVAD implantation has revolutionized the management of advanced heart failure, transforming the prognosis of patients once limited to transplant candidacy or palliative therapy.^1^ While survival benefits are well-established,^2^ functional and psychosocial outcomes remain equally critical determinants of post-implant quality of life.^3^ It is consistently associated with significant improvements in physical function, symptom burden, and patient-reported quality of life for patients with advanced heart failure, supporting its role in both prolonging survival and enhancing daily living and psychosocial well-being. These benefits are most pronounced with newer-generation devices and in well-selected patient populations, though adverse events and complications remain important considerations.^4^ Despite the mentioned gains of survival and improvement in quality of life with LVADs, there remains a delay in referring suitable patients to LVAD centers due in part to a lack of awareness about the quality of life gains that are related to LVAD use.^5^

Much of the existing functional and patient-reported outcome data in this field derive from earlier-generation continuous-flow devices or from large registry and clinical-trial populations treated at high-volume academic centers. The present study adds to this literature by reporting contemporary, real-world outcomes from an exclusively HeartMate 3 cohort managed in a community hospital setting, in patients who received the device predominantly as destination therapy (95%) and were largely INTERMACS profile II–III at implantation. By pairing several complementary, validated instruments—the KCCQ-12, EQ-5D-3L, EQ-VAS, and 6MWD—with patient-reported psychosocial measures such as health-related stress, treatment satisfaction, social engagement, and cognitive performance, it provides a multidimensional characterization of one-year recovery in precisely the type of patient and care setting that is increasingly common yet under-represented in the current functional-recovery literature.

## Methods

### Study design and population

This was a retrospective, single-center cohort of 41 consecutive adults implanted with HeartMate 3 LVADs between February 2022 and October 2024 at Bethesda North Hospital (a 440-bed community hospital serving the Greater Cincinnati Region) and Thomas Comprehensive Care Center (an ambulatory care facility with special emphasis on cardiovascular care). De-identified data were abstracted from the EMR using standardized forms per the protocol.

Participants’ inclusion criteria included age ≥18 years, HeartMate 3 implantation during the study window, and continued post-implant follow-up. Exclusion criteria mainly included pregnancy or incomplete records. A total of 41 patients were identified during the study period. All of these patients were managed at Bethesda North Hospital and the Thomas Comprehensive Care Center by providers specializing in advanced heart failure management. Follow-up duration was 2 years ± 0.3 years. Each patient was followed by specialized LVAD coordinators, who are trained nurses who provided ongoing counseling and education before and after LVAD implantation, in addition to a dedicated heart failure specialist.

All surveys and functional assessments were conducted before LVAD implantation, and a follow-up survey was administered at 12 months.

Of the 41 included patients, 35 had sufficiently complete paired pre- and post-implant data for the functional and patient-reported outcome analyses. Six patients were excluded from these paired analyses because KCCQ, 6-minute walk test, and/or quality-of-life forms were partially incomplete.

### Variables and definitions

Captured domains included demographics, comorbidities, device and peri-procedural factors (INTERMACS profile at time of implantation), and outcome-related measurements.

### Outcomes

Primary endpoints included changes from baseline to 12 months post LVAD implantation in multiple functional, quality of life, psychosocial measurements, like: Kansas City Cardiomyopathy Questionnaire-12 (KCCQ-12), EQ-5D-3L domains, EQ-VAS, 6-minute walk distance (6MWD), and patient-reported stress and satisfaction scores (0–10 scale).

Secondary outcomes included rates of LVAD driveline infection, all-cause mortality, and stroke.

### Statistical analysis

Data were analyzed using paired t-tests (or Wilcoxon signed-rank tests where appropriate). Descriptive comparisons used t-tests and chi-square (χ²) tests as appropriate. Variables with p < 0.05 were considered statistically significant. No imputation was performed for missing data; complete-case paired analyses were used for functional and patient-reported outcomes.

### Ethical considerations

The study was approved by the institutional review board with a waiver of consent due to retrospective design.

## Results

### Baseline characteristics of the patients

The overall cohort included 41 patients with LVADs, with a mean age of 62.2 ± 11 years. The majority of patients were Caucasian/White (68.3%), and 31.7% were African American. Females comprised 31.7% of the cohort. Cardiovascular comorbidities were common, including hypertension in 70.7%, coronary artery disease in 53.7%, diabetes mellitus in 51.2%, and atrial fibrillation or flutter in 56.1% of patients. Chronic kidney disease was present in 39.0%, while chronic obstructive pulmonary disease affected 26.8% of the cohort. The mean body mass index was 27.6 ± 5.4 kg/m². With respect to medication and lifestyle factors, 24.4% of patients were receiving insulin therapy. Alcohol use was reported in 17.1% of patients. A history of tobacco exposure was prevalent, with 56.1% being former smokers, 9.8% active smokers, and 34.1% never smokers. All patients had HeartMate 3 (HM3) LVADs, and the majority of patients (66%) had longstanding heart failure (>5 years) and received LVADs primarily as destination therapy (95%). The majority (80%) were INTERMACS class II–III at the time of implantation (Table 1). Paired analyses included 35 patients with complete evaluable pre/post data.

**Table 1:** Baseline Characteristics

**Characteristic**	**Overall Cohort (N = 41)**
Age, mean±SD (years)	**62.2±11**
Race, n (%)	
African American	13 (31.7)
Caucasian/White	28 (68.3)
Sex, n (%)	
Female	13 (31.7)
Male	28 (68.3)
Diabetes mellitus	21 (51.2)
Hypertension	29 (70.7)
Cirrhosis	2 (4.9)
Atrial fibrillation/flutter	23 (56.1)
COPD	11 (26.8)
Peripheral arterial disease	3 (7.3)
Malignancy	3 (7.3)
Chronic kidney disease	16 (39.0)
Pulmonary embolism	8 (19.5)
Obstructive sleep apnea	4 (9.8)
Coronary artery disease	22 (53.7)
BMI, mean±SD (kg/m²)	**27.6±5.4**
Alcohol use	7 (17.1)
Smoking status, n (%)	
Active smoker	4 (9.8)
Former smoker	23 (56.1)
Never smoker	14 (34.1)
Heart Failure Duration, n (%)	
< 1 year	3 (7.3)
1–5 years	11 (26.8)
>5 years	27 (65.9)
LVAD Indication, n (%)	
Bridge to transplant	2 (4.9)
Destination therapy	39 (95.1)
INTERMACS Profile, n (%)	
I	2 (4.9)
II	8 (19.5)
III	25 (61.0)
IV	4 (9.8)
V	1 (2.4)

### KCCQ-12 and functional improvement

**Figure 1:**
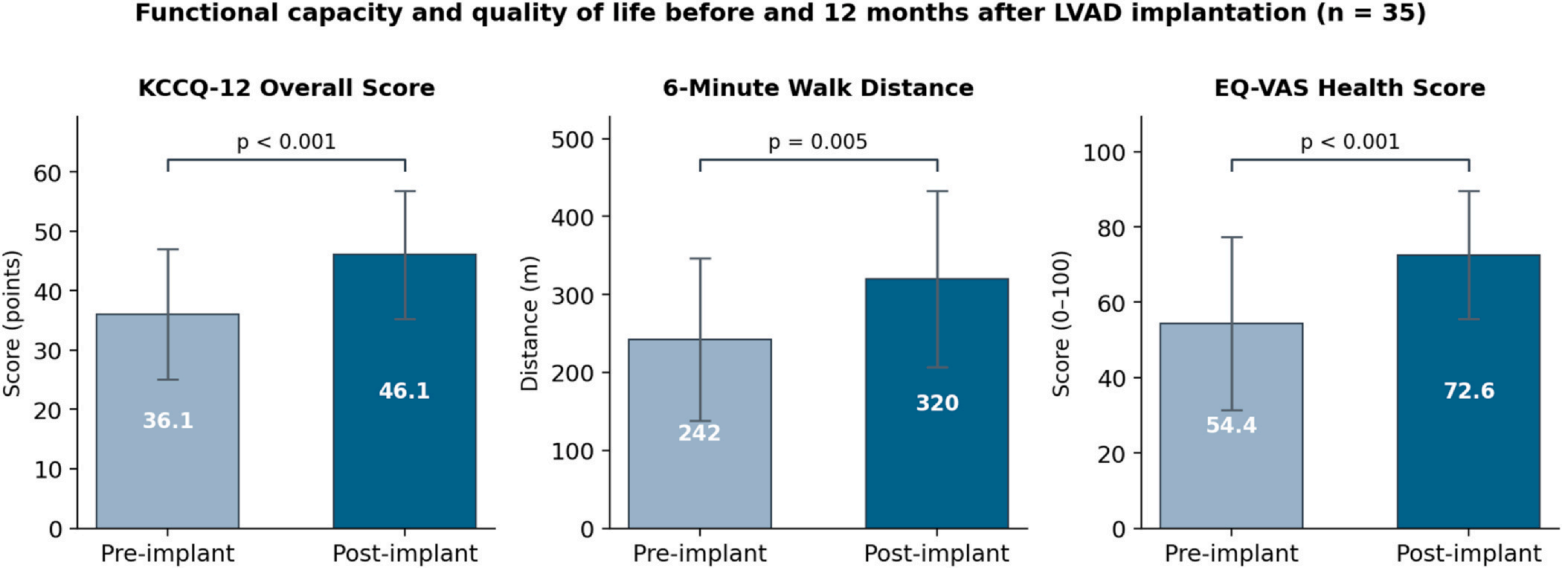
Pre- and post-implant comparison of primary outcomes. Functional capacity and health-related quality of life before and 12 months after LVAD implantation (paired cohort, n = 35). Bars represent mean values and error bars represent standard deviations for the Kansas City Cardiomyopathy Questionnaire-12 (KCCQ-12) overall score, the 6-minute walk distance, and the EuroQol Visual Analogue Scale (EQ-VAS). All three measures improved significantly from baseline to 12 months.

**Figure 2:**
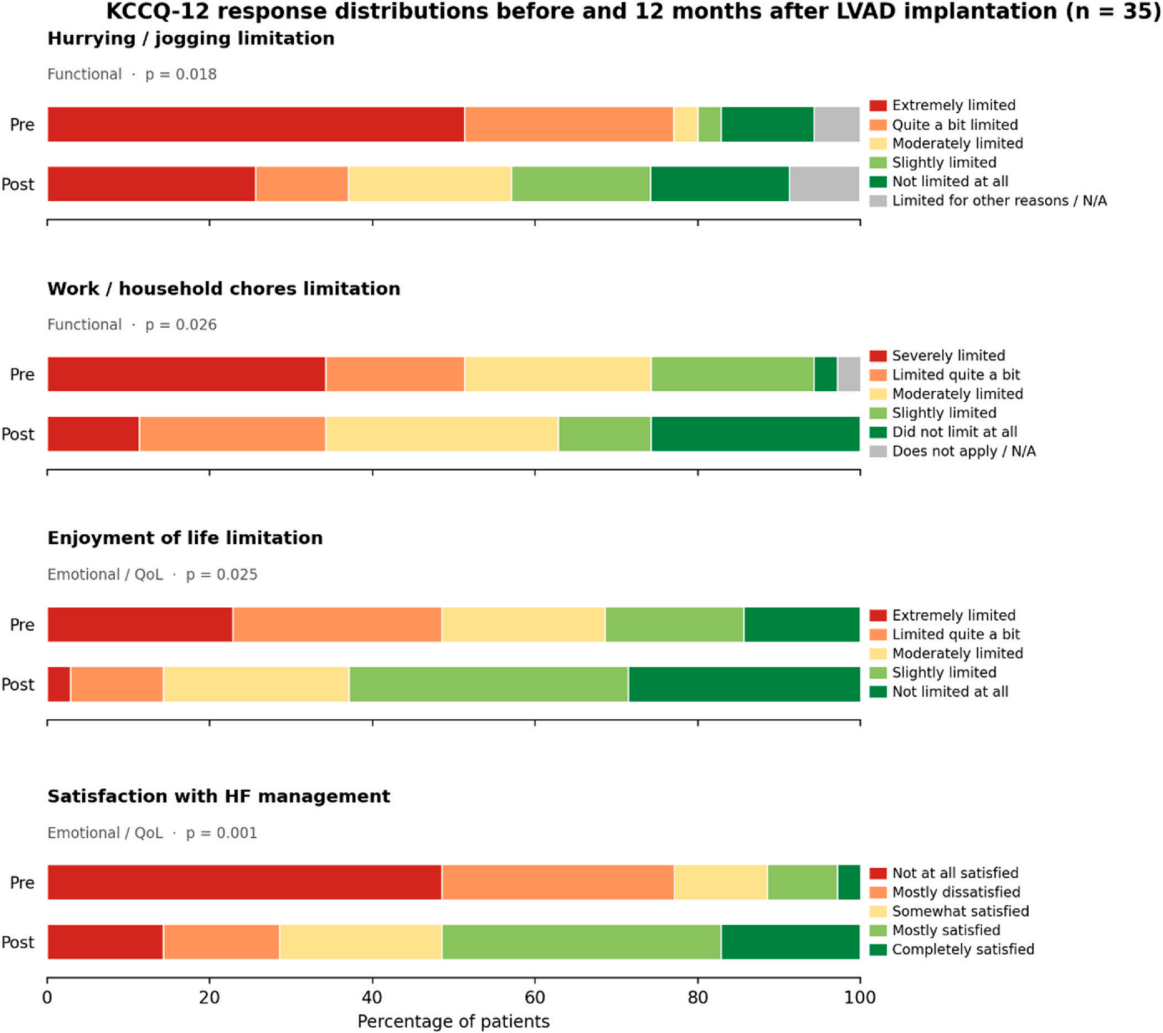
Pre- and post-implant comparison of patient-reported measures. KCCQ-12 response distributions before and 12 months after LVAD implantation (n = 35) for the four domains with statistically significant change: two functional (hurrying/jogging and work/household-chores limitation) and two emotional/quality-of-life (enjoyment of life and satisfaction with heart failure management). Segments are ordered from least to most favorable; the favorable (green) share increases from pre to post across all four domains.

At 12 months post-LVAD implantation, patients exhibited significant improvement in their total KCCQ-12 scores. It increased from 36.06 ± 10.98–46.06 ± 10.76 points (p < 0.001) with special improvements noted in the domains of physical limitations, satisfaction with heart failure management, enjoyment of life, and improved ability to do household chores (all p < 0.05) (Table 2). Another parameter related to function was the 6-minute walk test distance (6MWD), which improved by approximately 78 m (from 242 ± 104 m to 320 ± 113 m; p = 0.0054) (Figure 1). KCCQ12 important domain changes were highlighted in Figure 2.

**Table 2:** *Kansas City Cardiomyopathy Questionnaire–12 (KCCQ-12) Domains Before and After LVAD Implantation

**Measure**	**Response Level**	Pre-Implant (n=35)	Post-Implant (n=35)	**P value**
Physical Limitation				
Hurrying or jogging limitation, n (%)	Extremely limited	18 (51.4)	9 (25.7)	**0.018**
	Quite a bit limited	9 (25.7)	4 (11.4)	
	Moderately limited	1 (2.9)	7 (20.0)	
	Slightly limited	1 (2.9)	6 (17.1)	
	Not limited at all	4 (11.4)	6 (17.1)	
	Limited for other reasons / N/A	2 (5.7)	3 (8.6)	
Symptom Frequency				
Morning swelling frequency, n (%)	Every morning	7 (20.0)	2 (5.7)	0.117
	3+ times/week (not daily)	2 (5.7)	2 (5.7)	
	1–2 times/week	6 (17.1)	2 (5.7)	
	Less than once/week	9 (25.7)	9 (25.7)	
	Never (past 2 weeks)	11 (31.4)	20 (57.1)	
Fatigue frequency, n (%)	All the time	6 (17.1)	0 (0.0)	0.054
	Several times/day	6 (17.1)	4 (11.4)	
	At least once/day	4 (11.4)	5 (14.3)	
	3+ times/week	4 (11.4)	10 (28.6)	
	1–2 times/week	5 (14.3)	6 (17.1)	
	Less than once/week	7 (20.0)	3 (8.6)	
	Never (past 2 weeks)	3 (8.6)	7 (20.0)	
Shortness of breath frequency, n (%)	All the time	4 (11.4)	0 (0.0)	0.366
	Several times/day	2 (5.7)	4 (11.4)	
	At least once/day	3 (8.6)	1 (2.9)	
	3+ times/week	5 (14.3)	4 (11.4)	
	1–2 times/week	5 (14.3)	6 (17.1)	
	Less than once/week	6 (17.1)	6 (17.1)	
	Never (past 2 weeks)	10 (28.6)	14 (40.0)	
Orthopnea frequency, n (%)	Every night	6 (17.1)	6 (17.1)	0.294
	3+ times/week	3 (8.6)	2 (5.7)	
	1–2 times/week	4 (11.4)	1 (2.9)	
	Less than once/week	4 (11.4)	1 (2.9)	
	Never (past 2 weeks)	18 (51.4)	25 (71.4)	
Quality of Life				
Enjoyment of life limitation, n (%)	Extremely limited	8 (22.9)	1 (2.9)	**0.025**
	Moderately limited	7 (20.0)	8 (22.9)	
	Limited quite a bit	9 (25.7)	4 (11.4)	
	Slightly limited	6 (17.1)	12 (34.3)	
	Not limited at all	5 (14.3)	10 (28.6)	
Satisfaction with HF management, n (%)	Not at all satisfied	17 (48.6)	5 (14.3)	**0.001**
	Mostly dissatisfied	10 (28.6)	5 (14.3)	
	Somewhat satisfied	4 (11.4)	7 (20.0)	
	Mostly satisfied	3 (8.6)	12 (34.3)	
	Completely satisfied	1 (2.9)	6 (17.1)	
Social Limitation				
Effect on hobbies/recreation, n (%)	Did not limit at all	1 (2.9)	8 (22.9)	0.082
	Slightly limited	5 (14.3)	5 (14.3)	
	Limited quite a bit	9 (25.7)	6 (17.1)	
	Moderately limited	8 (22.9)	11 (31.4)	
	Severely limited	11 (31.4)	5 (14.3)	
	Does not apply / N/A	1 (2.9)	0 (0.0)	
Effect on work/household chores, n (%)	Did not limit at all	1 (2.9)	9 (25.7)	**0.026**
	Slightly limited	7 (20.0)	4 (11.4)	
	Limited quite a bit	6 (17.1)	8 (22.9)	
	Moderately limited	8 (22.9)	10 (28.6)	
	Severely limited	12 (34.3)	4 (11.4)	
	Does not apply / N/A	1 (2.9)	0 (0.0)	
Effect on visiting family/friends, n (%)	Did not limit at all	7 (20.0)	17 (48.6)	0.150
	Slightly limited	4 (11.4)	5 (14.3)	
	Limited quite a bit	7 (20.0)	4 (11.4)	
	Moderately limited	8 (22.9)	4 (11.4)	
	Severely limited	8 (22.9)	5 (14.3)	
	Does not apply / N/A	1 (2.9)	0 (0.0)	
KCCQ-12 Overall Score, mean±SD		36.06±10.98	46.06±10.76	**<0.001**

* Analyses are based on patients with complete paired data (n=35); 6 patients were excluded because of partially missing outcome forms.

### Quality-of-Life questionnaire and the EQ-5D-3L survey

Multiple aspects related to quality of life were assessed via a standard survey administered in the follow-up appointment post LVAD implantation in addition to the standardized EQ-5D-3L survey.

The majority of patients were either retired (40%) or too sick to work, with 14% being either actively working with steady employment or at least keeping house. There were no major changes in the primary daily physical activity before and after LVAD implantation. Social interactions were assessed, and on average, the mean number of close contacts that the patient interacted with on a monthly basis was around 10 ± 8.5, and it did not change significantly after LVAD implantation.

Almost one-third of patients reported unintentional weight loss of more than 10 pounds within the last 12 months prior to LVAD implantation. This proportion decreased to 14% after LVAD implantation, but the change did not reach statistical significance. Two notable outcomes that showed significant improvement were health-related stress (decreased from 6.4 to 4.4, p = 0.002) and satisfaction with current heart failure management over the last 3 months (increased from 7.2 to 8.5, p = 0.03) (Table 3).

**Table 3:** *Functional Status, Quality of Life, and EQ-5D-3L Measures Before and After LVAD Implantation

**Measure**	Pre-Implant (n=35)	Post-Implant (n=35)	**P value**
*Quality of Life Related Questions*			
Primary daily activity, n (%)			**0.782**
Actively working	5 (14.3)	5 (14.3)	
Keeping house	5 (14.3)	5 (14.3)	
Retired	14 (40.0)	13 (37.1)	
Too sick to work (disabled)	9 (25.7)	12 (34.3)	
Unknown	1 (2.9)	0 (0.0)	
Number of close contacts per month, mean±SD	10.06±8.54	9.74±9.60	0.886
Unintentional weight loss >10 lb, n (%)			0.078
No	22 (62.9)	30 (85.7)	
Yes	12 (34.3)	5 (14.3)	
Health-related stress (last month), mean±SD	6.44±2.94	4.43±2.32	**0.002**
Health-related coping (last month), mean±SD	6.82±2.47	7.80±1.92	0.071
Confidence managing HF, mean±SD	8.56±1.97	8.54±1.67	0.971
Satisfaction with HF therapy (last 3 months), mean±SD	7.24±2.69	8.46±1.93	**0.033**
Trail Making Test time (seconds), mean±SD	195.78±109.92	168.17±96.36	0.277
*EQ-5D-3L Domains*			
Mobility, n (%)			0.476
Confined to bed	4 (11.4)	2 (5.7)	
No problems walking	16 (45.7)	21 (60.0)	
Some problems walking	14 (40.0)	12 (34.3)	
Self-care, n (%)			0.323
No problems	19 (54.3)	25 (71.4)	
Some problems	14 (40.0)	10 (28.6)	
Unable	1 (2.9)	0 (0.0)	
Ability to carry out usual activities, n (%)			0.277
No problems	12 (34.3)	17 (48.6)	
Some problems	16 (45.7)	16 (45.7)	
Unable	6 (17.1)	2 (5.7)	
Reported pain/discomfort, n (%)			0.710
None	17 (48.6)	15 (42.9)	
Moderate	16 (45.7)	19 (54.3)	
Severe	1 (2.9)	1 (2.9)	
Reported Anxiety/depression, n (%)			0.145
None	15 (42.9)	17 (48.6)	
Moderate	15 (42.9)	18 (51.4)	
Severe	4 (11.4)	0 (0.0)	
*EQ-VAS health score, mean±SD*	54.38±22.95	72.57±17.04	**<0.001**

* Analyses are based on patients with complete paired data (n=35); 6 patients were excluded because of partially missing outcome forms.

**Table 4:** **Secondary Outcomes for the Total 41 Patients

**Outcome**	**Events (n)**	**Incidence (%)**
Stroke	5	12.2
LVAD driveline infection (DLI)	11	26.8
All-cause mortality	8	19.5
LVAD Removal	1	2.4

** Event rates are reported for the full cohort of 41 patients over the available follow-up period.

All of the EQ-5D-3L survey domains changed and trended favorably but did not reach significance. However, the EQ-VAS significantly improved by 18 points (from 54.38 ± 22.95–72.57 ± 17.04; p < 0.001). Changes in EQ-VAS, health-related stress, and satisfaction with therapy are illustrated in Figure 1. Cognitive follow-up testing included the Trail Making Test; performance trended toward improvement but did not reach statistical significance (Table 3).

### Secondary outcomes

Other observational secondary outcomes from the cohort were a stroke incidence rate of 12.2%, LVAD driveline infection rate of 26.8%, and all-cause mortality by the end of the follow-up period of 19.5%. One patient underwent LVAD removal because of a complicated driveline infection.

## Discussion

Although LVAD implantation is associated with improved survival, qualitative data demonstrate that patients continue to experience substantial, often under-recognized functional, psychosocial, and lifestyle limitations that shape their daily lives. Domains such as physical capacity, ability to work, social engagement, emotional well-being, and device-related burdens are not fully captured by traditional clinical outcomes, underscoring the importance of validated functional and patient-reported assessment tools to more comprehensively evaluate the impact of LVAD therapy.^6^

Tools that are used to assess functional and quality of life improvement in LVAD recipients are numerous, but some are more well-validated than others. KCCQ-12 is a validated, abbreviated patient-reported outcome measure for heart failure that maintains the psychometric properties of the original 23-item KCCQ, with strong evidence supporting its use for prognostication, clinical monitoring, and research.^7^ Its prognostic accuracy for outcomes such as death, hospitalization, and advanced therapies is comparable to the full KCCQ and superior to other heart failure-specific questionnaires like the Minnesota Living With Heart Failure Questionnaire.^8^ The KCCQ-OS score is the dominant predictor of 90-day hospitalizations and cumulative mortality, outperforming other clinical, demographic, and laboratory variables in machine learning models.^9^ Our cohort’s patients demonstrated significant improvement after 12 months in their KCCQ12 scores compared to their baseline (36.06 ± 10.98–46.06 ± 10.76 points, P < 0.001). This improvement in the mean is similar to previously reported improvements in KCCQ12 scores in HMII cohorts^3^ and is therefore consistent with the use of newer generations of LVAD devices. All components of the KCCQ12 score showed trends of improvement, but significance was mainly noted in improvement in previously noted physical limitations and ability to do house chores, improved satisfaction with heart failure management, and increased enjoyment of life activities. This is of significant value as many studies showed that heart failure patients suffer from dissatisfaction with their disease management^10^ that had improved after LVAD implantation in some studies compared to patients who elected to stay on optimal medical therapy.^11^ Of note, persistent dissatisfaction with heart failure management after LVAD was tied to poorer global outcomes and quality of life.^12^ Our cohort had initially around 48% “not at all satisfied” with improvement to 14% after 12 months post LVAD, while the percentage of patients who reported complete satisfaction or mostly satisfied improved to 51% (Table 2).

Another widely used tool is the EQ-5D-3L instrument that measures health-related quality of life (QOL) across five dimensions: mobility, self-care, usual activities, pain/discomfort, and anxiety/depression, each with three response levels (no problems, some problems, severe problems), it has the added benefit of examining some domains not covered by the KCCQ12 like anxiety and depression associated with heart failure or revealing unusual problems not typically associated with HF like pain.^13^ Measurement properties of the EQ-5D-3L are generally strong, with low rates of missing data (<3%), good construct and known-groups validity, and moderate to strong correlations with physical and generic health measures.^14^ It performed well across different European countries,^15^ and age groups.^16^

A tool that was derived from the EQ-5D was the EuroQol Visual Analogue Scale (EQ-VAS). It self-assesses general health: it asks respondents to rate their health today on a vertical thermometer from 0 (worst health you can imagine) to 100 (best health you can imagine).^17^ This tool was sensitive to changes in symptoms and health status, and its scores correlate with disease activity and other quality-of-life measures, though the correlation with the EQ-5D index is moderate, but it could be better in summarizing the patient's health from their perspective.^18^

Over most of the domains of the EQ-5D-3L, there were trends of improvement, like decreasing reported rates of severe anxiety/depression, improved mobility, and ability to self-care, but those trends did not reach statistical significance, likely due to the smaller sample size and the poor baseline of the patients to begin with. Of note, the EQ-VAS showed a significant improvement of a mean of 18 points compared to baseline (from 54.38 ± 22.95–72.57 ± 17.04, P value < 0.001) (Table 3).

Another method of monitoring functional improvement was measuring a six-minute walk test (6MWT). It is a validated, practical tool for assessing functional improvement, particularly in patients with chronic cardiopulmonary and vascular diseases.^19^ It provides an objective measure of submaximal exercise capacity that reflects daily activity tolerance and is sensitive to changes following therapeutic interventions such as rehabilitation, medical therapy, or exercise programs.^20^ It has been used as a tool for measuring functional improvements in post LVAD patients^21^ and was found to correlate with peak-VO2 on Cardiopulmonary exercise testing (CPET).^22^

The 6MWD in our cohort improved by a mean of approximately 78 m (from 242 ± 104 m to 320 ± 113 m; p = 0.0054), which carries potential implications for improved survival, as prior work has shown that in heart failure patients, a 6-minute walk distance of less than 300 m correlates with worse 3-year survival free of heart transplantation compared with distances greater than 300 m (62% vs 82%).^23^ This observation may carry greater weight in the context of the relatively sick baseline population in this cohort, with most patients in INTERMACS class II to III.

Because this was a retrospective study based on routinely collected clinical assessments, an LVAD-specific patient-reported outcome instrument such as the recently developed Mechanical Circulatory Support: Measures of Adjustment, Quality of Life, and Outcomes (MCS A-QOL) profile was not available.^24^ The observed driveline infection rate of 26.8% also underscores that meaningful functional recovery occurs alongside persistent device-related morbidity. This rate is broadly consistent with, though at the higher end of, contemporary reports, in which percutaneous driveline infections affect approximately 15–22% of recipients within the first one to two years and remain a leading cause of readmission. Beyond their procedural and microbiological burden, these infections carry prognostic weight, as registry data have linked percutaneous site infections to reduced survival.^25^ Because driveline infection imposes recurrent dressing changes, antibiotic courses, and exit-site discomfort, it is also a plausible detractor from patient-reported quality of life. Future studies should specifically examine whether infectious complications attenuate gains in KCCQ-12 scores and other patient-reported outcomes after LVAD implantation.

The added value of this study lies less in demonstrating that LVAD therapy improves outcomes—which prior work has already shown—and more in describing how recovery appears in a contemporary, predominantly destination-therapy HeartMate 3 cohort treated in a community hospital program. In addition to standard heart failure and generic quality-of-life measures, this analysis incorporated patient-reported stress, satisfaction with heart failure therapy, and cognitive follow-up testing, thereby providing a broader real-world picture of post-implant rehabilitation.

Overall, the functional, quality-of-life, and patient-experience improvements observed after LVAD implantation support the role of LVAD therapy in promoting meaningful recovery across multiple psychosocial and functional domains. Nonetheless, even with those measured improvements, HF patients with LVADs still have limitations in their overall function if compared with the general population, and this underscores the need for continued rehabilitation and integrating mental health and physical therapy programs within the LVAD programs.

## Limitations

Single-center retrospective design and modest sample size limit generalizability. Complete-case paired analyses may also introduce selection and survivor bias if patients with missing post-implant forms differed systematically from those included. Out of the 6 missing patients, one had a mortality event due to intracerebral bleeding following a motor vehicle accident unrelated to his LVAD morbidity. Furthermore, two patients had a driveline infection. None of the 6 patients had stroke. Because excluded patients could not be fully characterized retrospectively across all follow-up instruments, the effect of missingness on clinical event patterns could not be assessed directly. Other limitations include that missing follow-up data beyond 12 months limited the assessment of the persistence of the observed gains after LVAD implantation. Additionally, an LVAD-specific patient-reported outcome instrument was not available in this retrospective dataset. However, consistent trends across multiple instruments strengthen internal validity.

## Conclusion

At one-year post-LVAD implantation, patients demonstrated clinically and statistically significant gains in functional capacity and quality of life, alongside reduced stress and higher treatment satisfaction. These findings highlight the importance of comprehensive post-LVAD care that integrates physical rehabilitation and psychosocial support to optimize long-term outcomes.

## CRediT authorship contribution statement

Each author contributed substantially to the conception, analysis, drafting, and revision of this single-center cohort study and qualifies for authorship. All authors take responsibility for the contents of this manuscript. M.S., T.Z., M.A., O.J., K.O., P.T., and K.S. contributed to study conceptualization; M.S., S.S., T.Z., M.A., M.F., A.C., A.M., and S.K. contributed to data curation and analysis; and M.S., M.A., T.Z., and S.K. contributed to drafting the manuscript. All authors reviewed and approved the final version.

## Funding sources

This work did not receive any specific grant from funding agencies in the public, commercial, or not-for-profit sectors.

## Declaration of Competing Interest

The authors declare that they have no known competing financial interests or personal relationships that could have appeared to influence the work reported in this paper.
